# Don't Wear Your New Shoes (Yet): Taking the Right Steps to Become a Successful Principal Investigator

**DOI:** 10.1371/journal.pcbi.1002834

**Published:** 2013-01-31

**Authors:** Jeroen de Ridder, Thomas Abeel, Magali Michaut, Venkata P. Satagopam, Nils Gehlenborg

**Affiliations:** 1 Delft Bioinformatics Lab, Delft University of Technology, Delft, The Netherlands; 2 Netherlands Bioinformatics Centre, The Netherlands; 3 Broad Institute, Cambridge, Massachusetts, United States of America; 4 VIB Department of Plant Systems Biology, Ghent University, Ghent, Belgium; 5 Department of Molecular Carcinogenesis, Netherlands Cancer Institute, Amsterdam, The Netherlands; 6 Structural and Computational Biology Unit, European Molecular Biology Laboratory (EMBL), Heidelberg, Germany; 7 Luxembourg Centre for Systems Biomedicine (LCSB), University of Luxembourg, Campus Belval, House of Biomedicine, Luxembourg; 8 Harvard Medical School, Boston, Massachusetts, United States of America; Princeton University, United States of America

## Abstract

You finished your PhD, have been a postdoc for a while, and you start wondering, “What's next?” Suppose you come to the conclusion that you want to stay in academia, and move up the ladder to become a principal investigator (PI). How does one reach this goal given that academia is one of the most competitive environments out there? And suppose you do manage to snatch your dream position, how do you make sure you hit the ground running? Here we report on the workshop “P2P - From Postdoc To Principal Investigator” that we organized at ISMB 2012 in Long Beach, California. The workshop addressed some of the challenges that many postdocs and newly appointed PIs are facing. Three experienced PIs, **Florian Markowetz** (Group Leader, Cambridge Research Institute, Cancer Research UK), **Gary Bader** (Associate Professor, The Donnelly Centre, University of Toronto), and **Philip Bourne** (Professor, Skaggs School of Pharmacy and Pharmaceutical Sciences, University of California San Diego), provided insight into the transition from a trainee to PI and shared advice on how to make the best out it.

## Applying

And competitive it is! It turns out there are more brilliant postdocs and even much better qualified people looking for that same attractive principal investigator (PI) position you have laid your eyes on. You realize you are now your own “product”, and it is time to start selling, yet, how does one approach this?

Rule number one, according to Dr. Markowetz, is not to leapfrog the postdoc phase: “It is well worth the time spent as you extend your expertise, gain scientific maturity, and get the chance to switch fields.” As a postdoc, you can do your own research without all the responsibilities that come with running a lab. It is therefore not a good idea to do this in the same lab as you did your PhD; this is the time to explore new territory and learn to operate outside your comfort zone.

Albeit perhaps a “boring technicality of the job search”, a *concise* CV is nevertheless the first step towards a successful application. Four pages or below, well structured with clear headings and limited white space, Dr. Markowetz suggests. Moreover, don't underestimate the importance of tailoring your reference letters to the cultural preferences of the reader. A reference letter stating that your “research adheres to professional standards” is much less appreciated by a hiring authority in America than by one in Germany. The inverse is true for a letter stating that you were “the most awesome PhD student to ever graduate from the lab.” “Make sure you coach your referee in this,” Dr. Markowetz stresses.

One of the key components of your application for a PI position is a research statement. Using his own research statement from when he was applying for faculty positions, Dr. Markowetz showed how the desire to be complete and the overuse of buzzwords easily render such statements completely useless. A better statement is indeed much shorter and contains a specific research question. “Here, it is especially important that your statement stands out and is unique,” Dr. Markowetz emphasizes. “For the top jobs it's not enough to just do what everybody else is doing—they want to see that you have the potential to be a leader in the field. And the best way to do that is to show your individual view and ideas.”

Collecting all your “selling points” in your CV, reference letters, and mission statement is one thing, finding the right position is something completely different. Dr. Markowetz particularly points out the importance of exploring your own network: “get back in touch with all those people you know from your PhD lab, use LinkedIn (or similar networking tools) and check to see if you know someone from the inside that can get your CV to the top of the stack of hundreds of other CVs.” Moreover, letting others know you are looking for a job might put that good old grapevine to work, maybe even in a place you were not thinking of. Consequently, “thinking global” is key when identifying potential positions. “There is good science all over the globe, from the West and East Coasts of the United States and Canada, to Europe all the way through South Africa, South-East Asia, and Australia.”

## Interviewing

So, you were selected for an interview—are you nervous yet? You don't need to be if you do your homework well. According to Dr. Bader, “the formula for success is still prepare, prepare and prepare.” A typical interview visit lasts for one or two days and includes a seminar, a couple of meetings with heads of units and other PIs, and a dinner. On this subject Dr. Bader stresses: “Do engage in science, but stay away from politics. Also, wait for the proper time to discuss salary and other package details; they'll broach the topic at some point and that's the right time.”

It is important that you convince the other PIs in the department that you are the perfect collaborator. This is achieved by illustrating how your proposed research is complementary to theirs. You will likely meet the institute director or department chair, who will typically ask you how you are planning to start your group. “Again,” says Dr. Bader, “you better be prepared for this question and plan this in your mind ahead of time. What do you need to do your science: people, space, other resources? One helpful question you could ask yourself is: What will I do in my first week of setting up a new group?”

Energetically and with a grin, Dr. Markowetz recommends the following regarding interviews: “Do not forget to enjoy the ride! You'll be taken out for dinner, and are expected to interact socially with the people that will be your peers. While this is not a free vacation, there is absolutely no reason why you should be stressed out about the process; they already think you are worthy of the position, otherwise they wouldn't have invited you over. So enjoy your time out!”

Finally, Dr. Markowetz offers perhaps the most important rule for a successful interview: “Do not wear new shoes!” It is crucially important that you feel comfortable, and with new shoes, the only thing on your mind during the entire day will be your hurting feet.

## Deciding

Deciding to go for either a university, a research institute, or a hospital is dependent on what you are looking for. Dr. Bader suggests that typically universities will have more students that can be recruited to help with research projects and better chances of tenure (the tenure track is good since it, quite literally, ensures that your career stays on track), but may have less funding. Research institutes generally offer a 3- to 5-year startup grant, good research facilities, and the possibility for external funding, whereas hospitals offer first-line access to patient samples, their records, and, often, a good budget.

The quality and size of the local scientific community, i.e., collaborators, is another important factor to consider. Dr. Bader asks: “In case you get students, what is their quality? Can you get a joint appointment in two or more departments? This may help in getting more students.” Keeping tabs on what other facilities are offered in the area, such as DNA sequencing and high performance computing and whether or not you need to share this infrastructure, may be of similar importance.

“Finally,” Dr. Bader advises, “to ensure the continued success of your lab, it is important to understand not just the startup package, but also to know whether there are good local, regional, and national funding possibilities that one can apply for.“

## Negotiation

Scientists are not destined to earn millions and become rich, even though some do. If money is your primary aim, better change professions. Before continuing, Dr. Bader first asks, “I presume all the prospective football coaches have left the room now? Alright, now we can discuss the real negotiations.” The most important point to remember is that you and the chair (or the search committee) are *on the same team*. They *want* you to succeed in setting up a successful lab that will contribute to human knowledge, publish many papers, and bring in millions of dollars in grant money.

Before commencing negotiations, the first thing to prepare is a startup budget for 2–3 years that should cover personnel, hardware, software, travel, and extras of around 10% per person. Do consider the cost of living in your negotiations, and if you don't know, ask for help to get a fair number. Often, there are benefits to cover moving costs and assistance in looking for a house or buying a house, so use those to the fullest extent. Even though your initial budget only covers the first few years, you may want to plan your growth: extra office space, extra people, time on shared resources. With a semi-serious face, Dr. Bader says, “I don't think I have to tell you that it is a very good idea to get everything that is negotiated and agreed upon in writing.”

## Hiring Your Own Team

Congratulations! You made it into a junior PI position, and you even got some money to employ one or two students. Of course, you intend to find and hire *the best* students possible, but how do you find the perfect candidate?

“You should bear in mind that you are an *unrecognized* PI,” Dr. Bourne starts, “and would still like to attract good applicants.” The solution is to be proactive in attracting talent. If you want people to be interested in your lab, “ask the big questions.” Moreover, make sure you get the word out that you have a position: job boards are a first stop, but don't forget to use your network and write e-mails to other labs that may have good candidates they couldn't hire. “Or e-mail me”, says Dr. Bourne with a broad smile, “I get a dozen applications per week!” He quickly adds, “But don't forget, the most important thing in any interaction is to be respectful to candidates. If they take the effort to apply for a position in your lab, take the effort to send a note back, even if it is standardized.”

After successfully attracting applicants, it is time to start separating the wheat from the chaff. Since science is such a competitive business, everyone has good grades and everyone has published. It is therefore important to initially look for something *out of the ordinary*. “So what stands out?” Dr. Bourne provides an example: “Done a startup. Have been in industry. Recorded a platinum album. Just about anything that you don't see everyday. More than just good science.” While these eye-catchers make candidates stand out, you should of course remember the classic metrics that still need to be there, such as what they published (journals and citations), what their scientific pedigree is, whether they bring their own funding, and if the skills match with those required in your lab.

“I always say: you are looking for someone who is a little crazy, but not certifiably nuts,” Dr. Bourne explains. A good way to get a better feel for the candidate is by talking to their references. You will soon notice that all reference letters will be good. After all, why else would the candidate put them there? Surely, reading between the lines, which takes some effort and skill, may get you a little extra. At the end of the day, however, you really need to pick up the phone and actually talk to the referees. Ask them probing questions to get a better grasp on how good your potential hire really is: “How does this person rank between all the people that went through your lab? How much did they contribute to the paper? What is your major criticism about this person? Whatever you do,” Dr. Bourne advises, “*never* hire anyone without talking to all the references.”

Even though the candidates may look perfect on paper and after talking to their references, it is ill-advised to hire somebody without actually meeting them. Flying them out for a one- or two-day interview is money well spent. This is also the perfect time to get feedback from the other people in your lab to see whether the new hire would fit in with the group. The new hire has to match the laboratory habits. For instance, some people prefer to work only at night, which may create a serious problem. “Don't forget that a lab is a dynamic entity, and a wrong person can really impact that negatively,” says Dr. Bourne with a knowing smile. Especially if you are just setting up your lab, this is particularly crucial and the decision on who to hire can really make or break your lab and your career as PI.

Finally, you have found the perfect hire. “Don't celebrate too early—you still need to hire them,” Dr. Bourne warns. He explains, “If the candidate says, ‘I am almost done with my thesis’, this should raise a huge red flag and you should not trust that. Secondly, there is ‘The Bureaucracy’; waiting a year for a visa to go through is not uncommon.”

## Supervising

At last, you hired someone and it was (scientific) love at first sight. But there is just one catch: how to supervise this person? Surely, the new PhD student or postdoc is bright, but how to motivate him after a setback or entice him to become more creative? It turns out to be much harder than you thought to stimulate a brilliant trainee to actually produce brilliant work.

Dr. Bourne thinks that supervising is a genetic trait rather than something that can easily be learned. Some people are really good at it, others are not. Nevertheless, he shares some invaluable tips that have worked well for him.

First of all, supervising must be tailored to the individual. “You have to accept,” Dr. Bourne offers, “that some of your people will need a lot of supervising while others work best if you leave them to their own devices with a little bit of support every so often.” In other words, as a supervisor, your job is to enable them to perform well. While for some that means staying out of their way, for others it may mean that you have to tell them what would be a good next step to try.

“Then there are the so-called bad situations,” Dr. Bourne continues. Whenever you have a bad situation on your hands you should actively work to bring it in the open and resolve it. Don't expect the people involved to see reason or the other's point of view on their own. It is your responsibility to smooth any wrinkles in the fabric of your lab. Whatever you do, don't put your head in the sand and hope it blows over. “It won't. It will fester until it explodes,” says Dr. Bourne.

In this regard, you can learn a lot from reading a book on management in general. This will teach you a lot that you can use in day-to-day lab management. “*Good to Great* by Jim Collins is one of my personal favorites,” Dr. Bourne reveals.

But, above all, supervising people boils down to trust. You have to act in a way that your students and postdocs can trust you to have their best interests at heart. Vice versa, you should be able to trust your students and postdocs to have the best interest of the lab at heart. This trust is crucial to keep a good working relationship. Do not jeopardize this trust by making promises you can't keep. “Treat your trainees as you treat yourself. After all, you are all part of the same team,” Dr. Bourne concludes.

## Final Words

Applying for a position, finding the right position, and hiring your first students and supervising them in the early stage of their career is all part of the deal when you climb the scientific career ladder and aspire to become a PI and lead a research group. While drawing from their own experience, all three speakers shared a wide range of practical advice on how to approach each of these steps. Apart from these vital tips, they made it very clear: there is no perfect recipe to further your scientific career. There is, however, one prerequisite: do great science.

## Further Resources

All slides of this meeting are available on the ISCB Student Council website (http://www.iscbsc.org). As a result of his presentation at the workshop, Dr. Markowetz has written two comprehensive blog entries on applying for a PI position (http://scientificbsides.wordpress.com/2012/08/05/from-postdoc-to-pi-ten-simple-rules-for-applying-part-1/ and http://scientificbsides.wordpress.com/2012/08/08/from-postdoc-to-pi-ten-simple-rules-for-applying-part-2/). Dr. Bourne has written many excellent articles about career development in academia and beyond that have been published in the *PLOS Computational Biology* 10 Simple Rules Collection (http://www.ploscollections.org/article/browseIssue.action?issue=info%3Adoi%2F10.1371%2Fissue.pcol.v03.i01).

